# The Importance of GLUT3 for *De Novo* Lipogenesis in Hypoxia-Induced Lipid Loading of Human Macrophages

**DOI:** 10.1371/journal.pone.0042360

**Published:** 2012-08-02

**Authors:** Lu Li, Bo Liu, Liliana Håversen, Emma Lu, Lisa U. Magnusson, Marcus Ståhlman, Jan Borén, Göran Bergström, Malin C. Levin, Lillemor Mattsson Hultén

**Affiliations:** Sahlgrenska Center for Cardiovascular and Metabolic Research, Institute of Medicine, Sahlgrenska Academy, University of Gothenburg, Gothenburg, Sweden; Indiana University School of Medicine, United States of America

## Abstract

Atherosclerotic lesions are characterized by lipid-loaded macrophages (foam cells) and hypoxic regions. Although it is well established that foam cells are produced by uptake of cholesterol from oxidized LDL, we previously showed that hypoxia also promotes foam cell formation even in the absence of exogenous lipids. The hypoxia-induced lipid accumulation results from increased triglyceride biosynthesis but the exact mechanism is unknown. Our aim was to investigate the importance of glucose in promoting hypoxia-induced *de novo* lipid synthesis in human macrophages**.** In the absence of exogenous lipids, extracellular glucose promoted the accumulation of Oil Red O-stained lipid droplets in human monocyte-derived macrophages in a concentration-dependent manner. Lipid droplet accumulation was higher in macrophages exposed to hypoxia at all assessed concentrations of glucose. Importantly, triglyceride synthesis from glucose was increased in hypoxic macrophages. GLUT3 was highly expressed in macrophage-rich and hypoxic regions of human carotid atherosclerotic plaques and in macrophages isolated from these plaques. In human monocyte-derived macrophages, hypoxia increased expression of both GLUT3 mRNA and protein, and knockdown of GLUT3 with siRNA significantly reduced both glucose uptake and lipid droplet accumulation. In conclusion, we have shown that hypoxia-induced increases in glucose uptake through GLUT3 are important for lipid synthesis in macrophages, and may contribute to foam cell formation in hypoxic regions of atherosclerotic lesions.

## Introduction

Lipid-loaded macrophages and regions of hypoxia are well-known characteristics of atherosclerotic plaques [1]. The lipids in macrophages are predominantly cholesteryl esters and triglycerides and are stored in cytosolic lipid droplets [2]. Although it is established that accumulation of these cytosolic lipid droplets results from increased intracellular levels of cholesterol derived from uptake of oxidized LDL [3], our previous results indicate that hypoxia may exacerbate lipid accumulation in macrophages even in the absence of lipoproteins [4]. We showed that the hypoxia-induced lipid accumulation in macrophages is a result of increased triglyceride biosynthesis and decreased â-oxidation [4], but the exact mechanism is unknown.

Both monocytes and macrophages express glucose transporters GLUT1 and GLUT3 [5], and a recent study showed that hypoxia increases glucose uptake in macrophages [6]. Both GLUT1 and GLUT3 have been shown to be regulated by HIF-1á [7]. Although GLUT1 is expressed at high levels in macrophage-rich areas of advanced atherosclerotic plaques, it is also abundant in normal arteries and GLUT1 protein levels do not appear to be increased by hypoxia [6]. By contrast, the abundance of GLUT3 is dramatically higher in atherosclerotic plaques compared with normal arteries [6], suggesting that GLUT3 expression may be promoted by hypoxia. However, the link between hypoxia-induced glucose uptake, GLUT3 expression and lipid accumulation remains to be determined.

In this study, we tested the hypothesis that hypoxia-induced triglyceride synthesis and lipid accumulation in human macrophages in the absence of lipoproteins is promoted by increased glucose uptake. Furthermore, we investigated the role of GLUT3 in this process.

## Materials and Methods

### Primary Human Macrophages

Buffy coats were obtained from the local blood bank at Sahlgrenska University Hospital, Gothenburg, and human mononuclear cells were isolated by centrifugation in a discontinuous gradient of Ficoll-Paque (Pharmacia). Cells were seeded at a density of 2×10^6^ cells/ml and differentiated into macrophages in Macrophage-SFM (Gibco) containing granulocyte macrophage colony stimulating factor for 3 days followed by 4 days in serum-free RPMI 1640 (containing 11 mmol/l glucose).

To test the effect of extracellular glucose, macrophages were incubated in RPMI containing 1 mmol/l glucose for 5 h and then incubated with the indicated concentration of glucose in hypoxic (1% O_2_) or normoxic (21% O_2_) conditions for 24 h.

**Figure 1:**
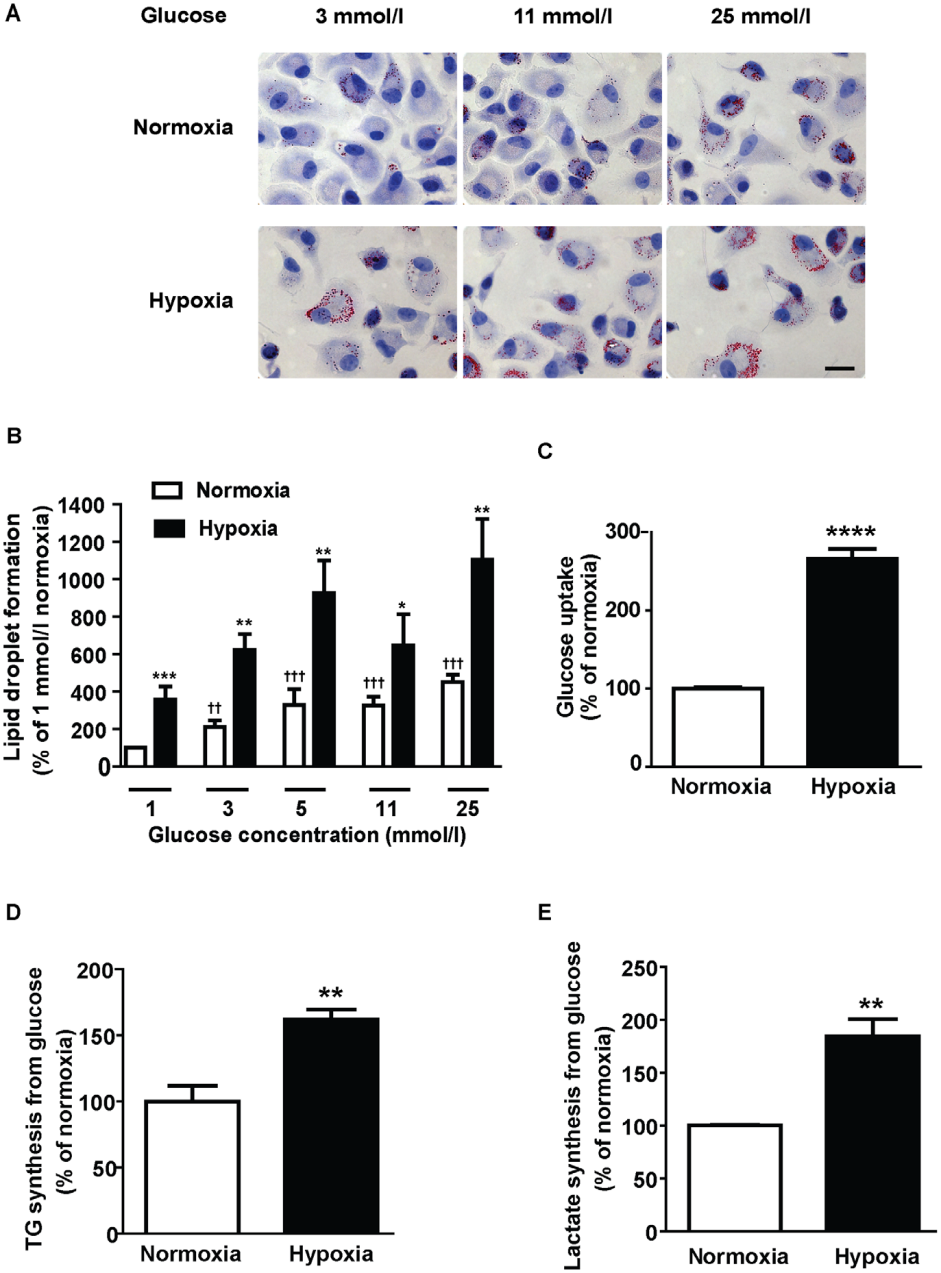
Hypoxia-induced increase in glucose uptake promotes accumulation of cytosolic lipid droplets in human macrophages through increased triglyceride synthesis. (A,B) Human monocyte-derived macrophages were cultured for 24 h in different glucose concentrations in the absence of exogenous lipids in normoxia (21% O_2_) or hypoxia (1% O_2_). (A) Micrograph of Oil Red O–stained macrophages exposed to the indicated glucose concentrations in normoxia or hypoxia. Scale bar 10 µm. (B) Quantification of (A). Data are mean ± SEM of all cells present in 20 randomly selected pictures from each of 7 macrophage donors. (C–E) Human monocytes-derived macrophages were incubated in medium containing 11 mmol/l glucose for 24 h in normoxia or hypoxia. (C) Glucose uptake. Data are mean ± SEM from 6 macrophage donors. (D) Triglyceride biosynthesis from radiolabeled glucose. Data are mean ± SEM from 7 macrophage donors. (E) Lactate biosynthesis from radiolabeled glucose. Data are mean ± SEM from 4 macrophage donors*P<0.05, **P<0.01, ***P<0.001, ****P<0.0001 vs normoxia at same glucose concentration; ††*P*<0.01, †††*P*<0.001 vs normoxia at 1 mmol/l glucose.

### Quantification of Oil Red O-stained Lipid Droplets

The total Oil Red O surface area was quantified as described in [8].

### Lipid Analysis of Isolated Lipid Droplets

Lipid droplets were isolated by gradient ultracentrifugation after nitrogen cavitation [9]. Lipids were extracted from droplets and quantified using straight-phase high-performance liquid chromatography with evaporative light scattering detection according to previous work [10].

### Glucose Uptake

Glucose uptake was measured as described [11]. Macrophages in 6-well plates were washed with uptake buffer containing 20 mmol/l Hepes, pH 7.4, 140 mmol/l NaCl, 5 mmol/l KCl, 1 mmol/l CaCl_2_ and 2.5 mmol/l MgSO_4_. Cells were then incubated with uptake buffer with 10 µmol/l deoxyglucose and 1 µCi/ml 2-deoxy-D-[2,6-^3^H]-glucose at 37°C. After 15 min, the cells were washed and harvested in 0.2 mol/l NaOH and the level of radioactivity was measured.

### Triglyceride Synthesis

Triglyceride synthesis was measured as described previously [12]. Briefly, macrophages were incubated for 24 h with serum-free RPMI 1640 containing 0.2 µCi/ml D-[1-^14^C]glucose (Amersham Biosciences). Cells were then washed three times with cold PBS and harvested in PBS. Lipids were extracted and triglycerides were separated with thin layer chromatography [13]. Finally, the level of radioactivity in the triglycerides was measured.

### Lactate Synthesis

Macrophages were incubated with 1.5 kBq/ml D-[^14^C(U)]glucose for 24 h. Lactate production was measured in the medium as described previously [14].

### Fatty Acid Synthase Activity

Fatty acid synthase activity was measured as incorporation of ^14^C-labeled malonyl-CoA into fatty acids as described [15].

### Atherosclerotic Plaques and Isolation of Plaque Macrophages

Carotid endarterectomies from patients with high-grade symptomatic carotid artery stenosis were obtained from the Göteborg and Umeå Atheroma Study Group biobank (http://www.wlab.gu.se/bergstrom/guvasc/). The study protocol was approved by the Ethical Committee of the University of Gothenburg (Dnr.404 - 09), and all subjects gave written informed consent.

For immunohistochemical studies, endarterectomies were fixed in formalin immediately after removal and embedded in paraffin blocks. Serial paraffin-embedded 5 µm sections were exposed to high-temperature antigen unmasking, and incubated with mouse monoclonal anti-human CD68 (1∶500; Novocastra Laboratories), anti-GLUT3 (1∶100; Abcam), and anti-HIF-1α (1∶400; Novus Biologicals). The stained sections were photographed using a Zeiss Mirax Scanner (Zeiss).

For isolation of plaque macrophages, endarterectomies from eight randomly selected patients were placed in Hank’s balanced salt solution immediately after removal. Aortic tissue from the carotid specimens was digested with collagenase as described [16], and the cells were suspended in RPMI 1640 medium supplemented with 1% bovine serum albumin. Macrophages were isolated by incubation with antibodies against the macrophage surface marker CD14 (BD Biosciences). The rosetting procedure was carried out by mixing pretreated cells with magnetic Dynabeads® coated with sheep anti-mouse IgG (www.invitrogen.com, Life Technologies), and the target cells were isolated with a magnet. Rosetted cells were washed several times with phosphate-buffered saline, and the absence of nonrosetted cells was confirmed by light microscopy. Cell viability assessed by trypan blue dye exclusion showed that 74.3% ±5.2% of CD14^+^ cells were viable. Total RNA was extracted from the isolated CD14^+^ cells.

### Analysis of Gene and Protein Expression

Total RNA was isolated with the RNeasy kit (Qiagen). cDNA was synthezised using the high capacity cDNA reverse transcription kit (Applied Biosystems) with random primers. mRNA expression of GLUT3 and actin was analyzed with TaqMan real-time PCR in an ABI PRISM 7700 sequence detection system (Applied Biosystems).

Immunoblots were prepared as described [11]. Total cellular lysates were prepared from human macrophages. Antibodies to GLUT3 and Tubulin were from Abcam.

### Immunocytochemistry

Immunostaining of GLUT3 with FITC-labeled antibodies (Abcam) and DAPI DNA stain for nuclear imaging in fluorescence microscopy was performed as recommended in [12].

### Transfection with GLUT3 siRNA

For GLUT3 knockdown experiments, primary macrophages were transfected with 20 nmol/l predesigned GLUT3 siRNA or negative control siRNA (Applied Biosystems) with HiPerfect transfection reagent (Qiagen) according to the manufacturers’ recommendation. After 24 h, cells were washed and siRNA was added again. Cells were then incubated in hypoxia for 24 h before analysis. To ensure the specificity of the knockdown, two different siRNA were used.

### Statistical Analysis

Data are shown as means ± SEM. Differences between groups were assessed with Student’s two-tailed t-test or one-way ANOVA. Graphpad Prism was used for statistical analysis.

**Figure 2:**
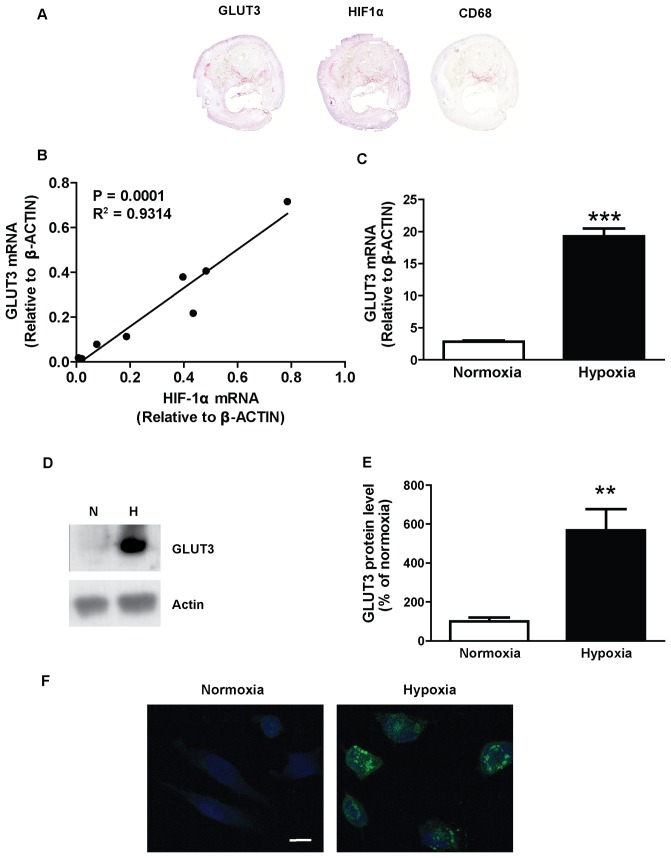
GLUT3 is abundant in macrophages isolated from human atherosclerotic plaques and in hypoxic human macrophages. (**A**) Representative immunohistochemical staining of sections of a human atherosclerotic carotid plaque with antibodies against GLUT3, HIF-1á and the macrophage marker CD68. (**B**) GLUT3 and HIF-1á mRNA expression in CD14^+^ macrophages isolated from 8 human atherosclerotic carotid plaques. (**C**–**F**) GLUT3 mRNA and protein levels in human monocyte-derived macrophages cultured in medium containing 11 mmol/l glucose for 24 h in normoxia (21% O_2_) or hypoxia (1% O_2_). (**C**) Real-time RT-PCR analyses of GLUT3 mRNA expression. Data are mean ± SEM from 6 macrophage donors. (**D**) Representative immunoblots of GLUT3 and tubulin in normoxic (N) and hypoxic (H) human macrophages. (**E**) Quantification of immunoblots. Data are mean ± SEM from 6 macrophage donors. (**F**) Representative immunoflourescent images of macrophages cultured in normoxia or hypoxia and stained for GLUT3 (green) and nuclei (blue). Scale bar 10 µm. **P<0.01, ***P<0.001 vs normoxia.

## Results and Discussion

We showed that extracellular glucose promoted accumulation of Oil Red O-stained lipid droplets in human monocyte-derived macrophages in the absence of exogenous lipids in a concentration-dependent manner (Fig. 1A,B). This effect was observed in macrophages incubated in both normoxic and hypoxic conditions, but the lipid droplet formation was higher in hypoxic macrophages at all assessed concentrations of glucose (Fig. 1B). These data show that lipid droplet formation is increased with higher extracellular glucose levels and suggest that hypoxia promotes increased lipid accumulation even in hyperglycemic conditions.

**Figure 3:**
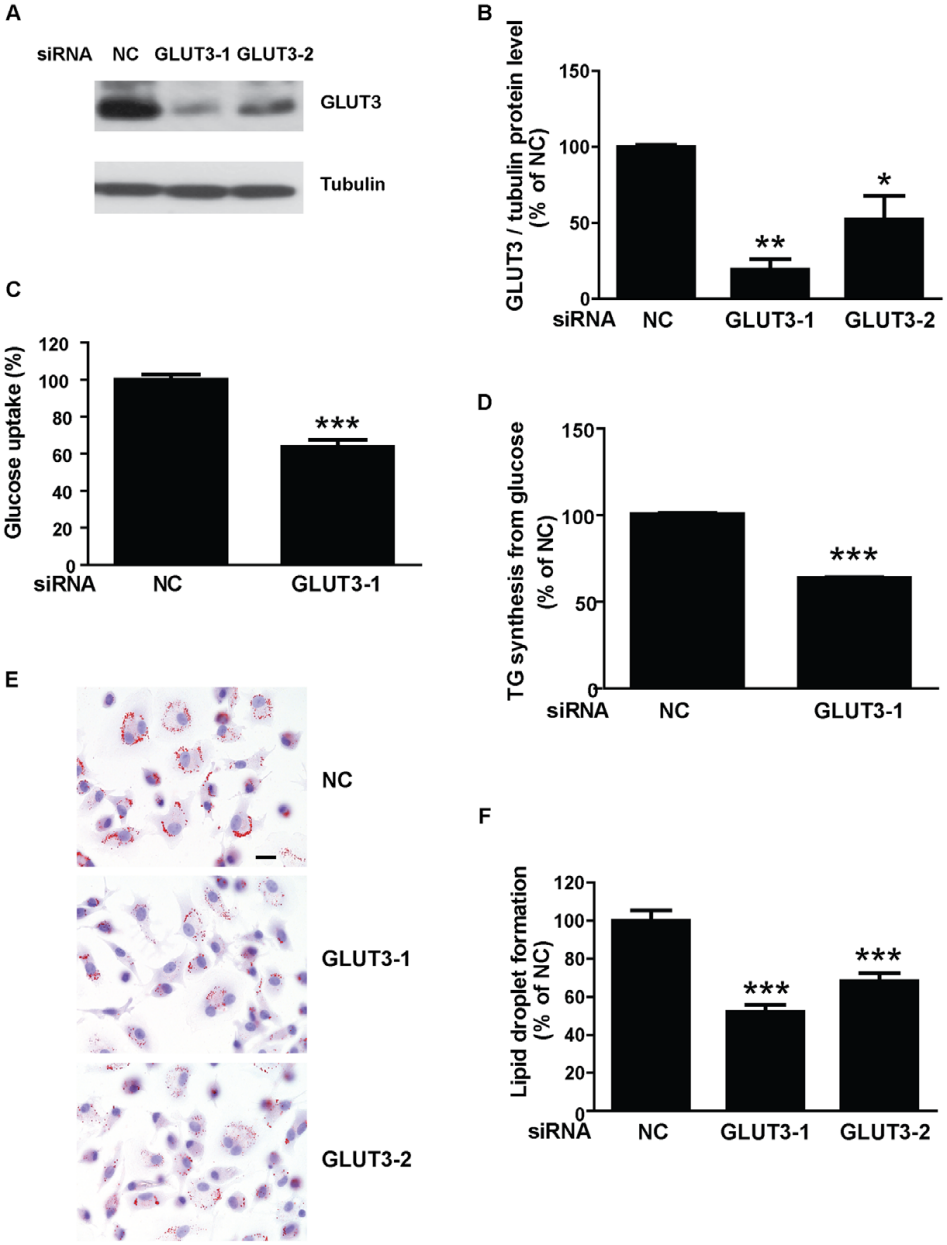
GLUT3 knockdown reduces glucose uptake and lipid droplet formation in hypoxic human macrophages. Human monocyte-derived macrophages were transfected with control siRNA or siRNA against GLUT3 and cultured in medium containing 11 mmol/l glucose for 24 h in hypoxia (1% O_2_). (**A**) Representative immunoblots of GLUT3 and tubulin in hypoxic human macrophages transfected with negative control (NC) or GLUT3 siRNA. (**B**) Quantification of (**A**). Data are mean ± SEM from 4 macrophage donors. (**C**) Knockdown of GLUT3 reduces glucose uptake in hypoxic human macrophages. Data are mean ± SEM from 6 macrophage donors. (**D**) Knockdown of GLUT3 reduces triglyceride biosynthesis from radiolabeled glucose in hypoxic human macrophages. Data are mean ± SEM from 3 macrophage donors. (**E**) Micrograph of Oil Red O-stained hypoxic macrophages transfected with control siRNA or GLUT3 siRNA. Scale bar 10 µm. (**F**) Quantification of (**E**). Data are mean ± SEM of all cells present in 20 randomly selected pictures from each of 4 macrophage donors. *P<0.05; **P<0.01; ***P<0.001 vs negative siRNA control.

We showed that lipid droplets isolated from human macrophages incubated in 25 mmol/L glucose at normoxia for 24 h were rich in triglycerides (70.4±7.2%) with low levels of cholesterol (19.8±6.8%) and cholesteryl ester (9.8±1.1%) (*n* = 4 macrophage donors). We have previously shown that the lipid droplets induced in response to hypoxia in human macrophages are promoted by increased cellular triglyceride levels with no changes in free cholesterol or cholesteryl ester at high glucose concentrations [4]. Furthermore, we showed increased glucose uptake in macrophages incubated in hypoxia (Fig. 1C), in agreement with earlier studies in human macrophages [6]. Importantly, we showed that hypoxia promoted increased triglyceride synthesis from radiolabeled glucose (Fig. 1D), which indicates that hypoxia-induced glucose uptake promotes lipid droplet formation in human macrophages by increasing triglyceride accumulation. Our data are thus in agreement with Parathath et al. who showed that hypoxia promotes significant triglyceride accumulation at both normal and high glucose concentrations [17].

Because it is well known that hypoxia also enhances glycolysis, we measured the incorporation of radiolabeled glucose into lactate in human macrophages. We observed that hypoxia promoted a 1.8-fold increase of the incorporation of glucose into lactate (Fig. 1E), which was similar to the 1.6-fold increase with hypoxia observed for the incorporation of glucose into triglycerides (Fig. 1D). Increased lactate concentration in hypoxic atherosclerotic plaques results in an acidic pH and promotes extracellular accumulation of lipoproteins [18].

There is very little earlier research showing the link between hypoxia-induced glucose uptake and triglyceride accumulation, but our data are in agreement with an early study showing that glucose is used for triglyceride synthesis in rabbit atherosclerotic aortas incubated in hypoxia [19]. Recent evidence suggests potentially important crosstalk between hypoxia and genes controlling lipogenesis, which results in enhanced lipid synthesis in macrophages [20]. We previously performed a DNA microarray analysis and observed a small increase in the mRNA expression of stearoyl-CoA desaturase (an enzyme involved in fatty acid synthesis) in macrophages exposed to hypoxia, but we did not observe any effects of hypoxia on fatty acid synthase, glycerol-3-phosphate acyltransferase or acylglycerol phosphate acyltransferase [4]. In our present study, we also analyzed fatty acid synthase activity, but did not show any changes in activity of this enzyme in macrophages exposed to hypoxia compared with normoxia (data not shown). We therefore do not have any clear evidence to show a role for increased levels or activity of lipogenesis enzymes in hypoxia-induced triglyceride accumulation.

An earlier study has shown that uptake of the glucose analog [^18^F]-fluorodeoxyglucose (^18^FDG) is higher in symptomatic compared with non-symptomatic carotid human lesions, with no measurable uptake in normal carotid arteries, and that ^18^FDG accumulates in macrophage-rich regions of the plaque [21]. This study suggests that regulation of the glucose uptake mechanisms differs depending on the severity of the lesion, but the details are not understood. It is known that GLUT1 is abundant in both normal arteries and in macrophage-rich regions of atherosclerotic plaques and does not appear to increase in response to hypoxia [6]. By contrast, GLUT3 is not detectable in normal arteries and increases dramatically in macrophage-rich regions of atherosclerotic plaques [6], suggesting that GLUT3 expression is induced by hypoxia.

Here, we verified that GLUT3 was abundant in macrophage-rich regions of human atherosclerotic plaques and showed that it was present in the same areas as the hypoxia marker HIF-1á (Fig. 2A). We also isolated macrophages from these plaques and confirmed the presence of GLUT3 mRNA (Fig. 2B). GLUT1, GLUT4 and GLUT5 mRNA levels were barely detectable (data not shown). Interestingly, GLUT3 mRNA levels correlated with HIF-1á mRNA levels (Fig. 2B). *In silico* analyses of the GLUT3 promoter indicated several potential binding sites for HIF-1á. Furthermore, an earlier analysis of GLUT3 promoter deletion sequences indicates that a putative hypoxia-response element is critical in GLUT3 promoter activity [22]. Thus, it is likely that increased GLUT3 expression in response to hypoxia is at least partially induced through a direct effect of HIF-1á.

We also investigated if hypoxia affected GLUT3 levels in human monocyte-derived macrophages. Indeed, we showed that hypoxia promoted a 7-fold increase in GLUT3 mRNA expression (Fig. 2C) and an 6-fold increase in GLUT3 protein levels (Figs. 2D,E). The increase in GLUT3 protein levels was confirmed by immunofluorescence (Fig. 2F). Thus, we have shown that hypoxia promotes increased GLUT3 levels in macrophages, which could contribute to increased glucose uptake in atherosclerotic plaques.

We next investigated whether the increased GLUT3 expression observed in response to hypoxia might play a role in promoting lipid accumulation. Knockdown of GLUT3 expression by siRNA in hypoxic macrophages reduced GLUT3 levels by 60–90% (Fig. 3A,B) and significantly reduced the uptake of 2-deoxy-D-[2,6-^3^H]-glucose (Fig. 3C), the incorporation of radiolabeled glucose into triglycerides (Fig. 3D) and the lipid droplet formation (Fig. 3E,F). These results indicate that glucose uptake through GLUT3 plays a major role in hypoxia-induced lipid accumulation in human macrophages.

In summary, we have shown the importance of glucose and GLUT3 in promoting hypoxia-induced *de novo* lipid synthesis in human monocyte-derived macrophages in the absence of lipoproteins. We showed that lipid accumulation increased with increasing glucose concentration and that the increased lipid formation was more pronounced in hypoxic conditions. As expected, hypoxia increased glucose uptake, and, importantly, we showed that triglyceride synthesis from glucose was increased in hypoxia. Analysis of human carotid endarterectomies demonstrated the abundance of GLUT3 in macrophage-rich and hypoxic regions of the plaques and, more specifically, in macrophages isolated from these plaques. Furthermore, in human monocyte-derived macrophages, we showed that hypoxia increased both GLUT3 mRNA and protein levels and that GLUT3 inhibition by siRNA significantly reduced both glucose uptake and lipid droplet accumulation.

We postulate that triglyceride-loaded macrophages indicate a potential link between the high levels of circulating glucose observed in patients with type 2 diabetes and the development of atherosclerosis. Indeed, vascular uptake of glucose is higher in patients with higher plasma glucose levels and is associated with vascular inflammation [23]. Furthermore, patients with type 1 diabetes generally have increased blood glucose levels in the absence of dyslipidemia, and a clinical study reported that the increased risk of cardiovascular complications in these patients can be reduced by improving glycemic control [24]. However, hyperglycemia alone has not been shown to accelerate atherosclerosis in animal models and it is likely that other factors such as basal lipid levels are involved [25]. Our results open up the possibility that lipid loading of macrophages is caused not only by endocytosis of LDL or modified LDL, but also by uptake of glucose and endogenous synthesis of triglycerides.

## References

[1] Hulten LM, Levin M (2009) The role of hypoxia in atherosclerosis. Curr Opin Lipidol 20: 409–414. 19644366 10.1097/MOL.0b013e3283307be8

[2] Martin S, Parton RG (2006) Lipid droplets: a unified view of a dynamic organelle. Nat Rev Mol Cell Biol. 10.1038/nrm1912 16550215

[3] Hansson GK, Robertson AK, Soderberg-Naucler C (2006) Inflammation and Atherosclerosis. Annu Rev Pathol Mech Dis 1: 297–329. 10.1146/annurev.pathol.1.110304.100100 18039117

[4] Bostrom P, Magnusson B, Svensson PA, Wiklund O, Boren J, et al (2006) Hypoxia converts human macrophages into triglyceride-loaded foam cells. Arterioscler Thromb Vasc Biol 26: 1871–1876. 16741148 10.1161/01.ATV.0000229665.78997.0b

[5] Fu Y, Maianu L, Melbert BR, Garvey WT (2004) Facilitative glucose transporter gene expression in human lymphocytes, monocytes, and macrophages: a role for GLUT isoforms 1, 3, and 5 in the immune response and foam cell formation. Blood Cells Mol Dis 32: 182–190. 14757434 10.1016/j.bcmd.2003.09.002

[6] Folco EJ, Sheikine Y, Rocha VZ, Christen T, Shvartz E, et al (2011) Hypoxia but not inflammation augments glucose uptake in human macrophages: Implications for imaging atherosclerosis with 18fluorine-labeled 2-deoxy-D-glucose positron emission tomography. J Am Coll Cardiol 58: 603–614. 21798423 10.1016/j.jacc.2011.03.044

[7] Semenza GL (2003) Targeting HIF-1 for cancer therapy. Nat Rev Cancer 3: 721–732. 13130303 10.1038/nrc1187

[8] Bostrom P, Rutberg M, Ericsson J, Holmdahl P, Andersson L, et al (2005) Cytosolic lipid droplets increase in size by microtubule-dependent complex formation. Arterioscler Thromb Vasc Biol 25: 1945–1951. 16051877 10.1161/01.ATV.0000179676.41064.d4

[9] Andersson L, Bostrom P, Ericson J, Rutberg M, Magnusson B, et al (2006) PLD1 and ERK2 regulate cytosolic lipid droplet formation. J Cell Sci 119: 2246–2257. 16723731 10.1242/jcs.02941

[10] Homan R, Anderson MK (1998) Rapid separation and quantitation of combined neutral and polar lipid classes by high-performance liquid chromatography and evaporative light-scattering mass detection. Journal of Chromatography B 708: 21–26. 10.1016/s0378-4347(97)00651-8 9653942

[11] Frost SC, Lane MD (1985) Evidence for the involvement of vicinal sulfhydryl groups in insulin-activated hexose transport by 3T3-L1 adipocytes. J Biol Chem 260: 2646–2652. 3882699

[12] Magnusson B, Asp L, Bostrom P, Ruiz M, Stillemark-Billton P, et al (2006) Adipocyte Differentiation-Related Protein Promotes Fatty Acid Storage in Cytosolic Triglycerides and Inhibits Secretion of Very Low-Density Lipoproteins. Arterioscler Thromb Vasc Biol. 10.1161/01.ATV.0000223345.11820.da 16627799

[13] Andersson M, Wettesten M, Boren J, Magnusson A, Sjoberg A, et al (1994) Purification of diacylglycerol:acyltransferase from rat liver to near homogeneity. J Lipid Res 35: 535–545. 8014588

[14] Horowicz P, Larrabee MG (1962) Metabolic partitioning of carbon from glucose by a mammalian sympathetic ganglion. J Neurochem 9: 407–420. 14448994 10.1111/j.1471-4159.1962.tb09468.x

[15] Brusselmans K, Vrolix R, Verhoeven G, Swinnen JV (2005) Induction of cancer cell apoptosis by flavonoids is associated with their ability to inhibit fatty acid synthase activity. J Biol Chem 280: 5636–5645. 15533929 10.1074/jbc.M408177200

[16] Mattsson L, Bondjers G, Wiklund O (1991) Isolation of cell populations from arterial tissue, using monoclonal antibodies and magnetic microspheres. Atherosclerosis 89: 25–34. 1663356 10.1016/0021-9150(91)90004-m

[17] Parathath S, Mick SL, Feig JE, Joaquin V, Grauer L, et al (2011) Hypoxia is present in murine atherosclerotic plaques and has multiple adverse effects on macrophage lipid metabolism. Circ Res 109: 1141–1152. 21921268 10.1161/CIRCRESAHA.111.246363 PMC3208906

[18] Oorni K, Kovanen PT (2006) Enhanced extracellular lipid accumulation in acidic environments. Curr Opin Lipidol 17: 534–540. 16960502 10.1097/01.mol.0000245259.63505.c2

[19] Howard CF Jr (1972) Aortic lipogenesis during aerobic and hypoxic incubation. Atherosclerosis 15: 359–369. 5051657 10.1016/0021-9150(72)90025-1

[20] Na TY, Lee HJ, Oh HJ, Huh S, Lee IK, et al (2011) Positive cross-talk between hypoxia inducible factor-1alpha and liver X receptor alpha induces formation of triglyceride-loaded foam cells. Arterioscler Thromb Vasc Biol 31: 2949–2956. 21940948 10.1161/ATVBAHA.111.235788

[21] Rudd JH, Warburton EA, Fryer TD, Jones HA, Clark JC, et al (2002) Imaging atherosclerotic plaque inflammation with [18F]-fluorodeoxyglucose positron emission tomography. Circulation 105: 2708–2711. 12057982 10.1161/01.cir.0000020548.60110.76

[22] Yu J, Li J, Zhang S, Xu X, Zheng M, et al (2012) IGF-1 induces hypoxia-inducible factor 1alpha-mediated GLUT3 expression through PI3K/Akt/mTOR dependent pathways in PC12 cells. Brain Res 1430: 18–24. 22104347 10.1016/j.brainres.2011.10.046

[23] Kim TN, Kim S, Yang SJ, Yoo HJ, Seo JA, et al (2010) Vascular inflammation in patients with impaired glucose tolerance and type 2 diabetes: analysis with 18F-fluorodeoxyglucose positron emission tomography. Circ Cardiovasc Imaging 3: 142–148. 20061516 10.1161/CIRCIMAGING.109.888909

[24] Nathan DM, Cleary PA, Backlund JY, Genuth SM, Lachin JM, et al (2005) Intensive diabetes treatment and cardiovascular disease in patients with type 1 diabetes. N Engl J Med 353: 2643–2653. 16371630 10.1056/NEJMoa052187 PMC2637991

[25] Kanter JE, Johansson F, LeBoeuf RC, Bornfeldt KE (2007) Do glucose and lipids exert independent effects on atherosclerotic lesion initiation or progression to advanced plaques? Circ Res 100: 769–781. 17395883 10.1161/01.RES.0000259589.34348.74

